# Adenosine in Interventional Cardiology: Physiopathologic and Pharmacologic Effects in Coronary Artery Disease

**DOI:** 10.3390/ijms25115852

**Published:** 2024-05-28

**Authors:** Enrico Marchi, Iacopo Muraca, Martina Berteotti, Anna Maria Gori, Renato Valenti, Rossella Marcucci

**Affiliations:** 1 Department of Experimental and Clinical Medicine, School of Human Health Sciences, Careggi University Hospital, University of Florence, 50134 Florence, Italy; 2 Division of Interventional Cardiology, Cardiothoracovascular Department, Careggi University Hospital, 50134 Florence, Italy; 3 Atherothrombotic Center, Department of Experimental and Clinical Medicine, University of Florence, AOU Careggi, 50134 Florence, Italyrossella.marcucci@unifi.it (R.M.)

**Keywords:** coronary artery disease, antiplatelet therapy, no-reflow phenomenon, stress testing

## Abstract

This review article focuses on the role of adenosine in coronary artery disease (CAD) diagnosis and treatment. Adenosine, an endogenous purine nucleoside, plays crucial roles in cardiovascular physiology and pathology. Its release and effects, mediated by specific receptors, influence vasomotor function, blood pressure regulation, heart rate, and platelet activity. Adenosine therapeutic effects include treatment of the no-reflow phenomenon and paroxysmal supraventricular tachycardia. The production of adenosine involves complex cellular pathways, with extracellular and intracellular synthesis mechanisms. Adenosine’s rapid metabolism underscores its short half-life and physiological turnover. Furthermore, adenosine’s involvement in side effects of antiplatelet therapy, particularly ticagrelor and cangrelor, highlights its clinical significance. Moreover, adenosine serves as a valuable tool in CAD diagnosis, aiding stress testing modalities and guiding intracoronary physiological assessments. Its use in assessing epicardial stenosis and microvascular dysfunction is pivotal for treatment decisions. Overall, understanding adenosine’s mechanisms and clinical implications is essential for optimizing CAD management strategies, encompassing both therapeutic interventions and diagnostic approaches.

## 1. Introduction

Adenosine is an endogenous purine nucleoside produced by the dephosphorylation of ATP (Adenosine TriPhosphate) and AMP (Adenosine MonoPhosphate). It is released mostly in the context of ischemia, beta-adrenergic stimulation, hypoxia, and inflammation [1,2]. It is also an exogenous drug with indications in both therapeutic, such as treatment of the no-reflow phenomenon or of paroxysmal supraventricular tachycardia, and diagnostic scenarios, such as in myocardial perfusion imaging.

Adenosine effects are mediated by specific GPCRs (G-Protein Coupled Receptors): A_1_R, A_2A_R, A_2B_R and A_3_R. The main effects of adenosine on the cardiovascular system effects are on vasomotor function and blood pressure regulation, heart rate, and platelet activity [3]. The aim of this review is to summarize the role of adenosine in diagnosis and treatment of coronary artery disease in both invasive and non-invasive scenarios.

## 2. Cellular Mechanism behind Adenosine Effects

### 2.1. Adenosine Production and Metabolism

The main source of endogenous adenosine is represented by endothelial and muscle cells, even though some level of adenosine synthesis can be found in most cells.

Adenosine production follows two main pathways: the extracellular one mediated by the hydrolysis of AMP, ADP (Adenosine DiPhosphate), and ATP and the intracellular one, through the hydrolytic metabolism of S-adenosyl-L-homocysteine (SAH).

The extracellular production of adenosine is enhanced by stressors and relies on specific enzymes: ectonucleoside triphosphate diphosphohydrolase (CD39) and ecto-5′-nucleotidase (CD73) [4]. The free passage of adenosine through the cell membrane is guaranteed by specific nucleoside transporters named concentrative nucleoside transporters (CNTs) and equilibrative nucleoside transporters (ENTs), and the direction of the adenosine flow is depended on its concentration between the intracellular and extracellular compartment [3].

The intracellular synthesis of adenosine is a consequence of hydrolysis of AMP and S-adenosyl-L-homocysteine mediated by CD73 and SAH hydrolase [5].

The fast catabolism of adenosine involves the deamination to inosine though adenosine deaminase (ADA) and the phosphorylation to AMP by adenosine kinase (AK) and its consequence is a physiological short half-life below 1 s [3]. Inosine is then converted into xanthine and finally to uric acid through xanthine oxidase (XO).

### 2.2. Adenosine Receptor and Effects on the Cardiovascular System

**Figure 1:**
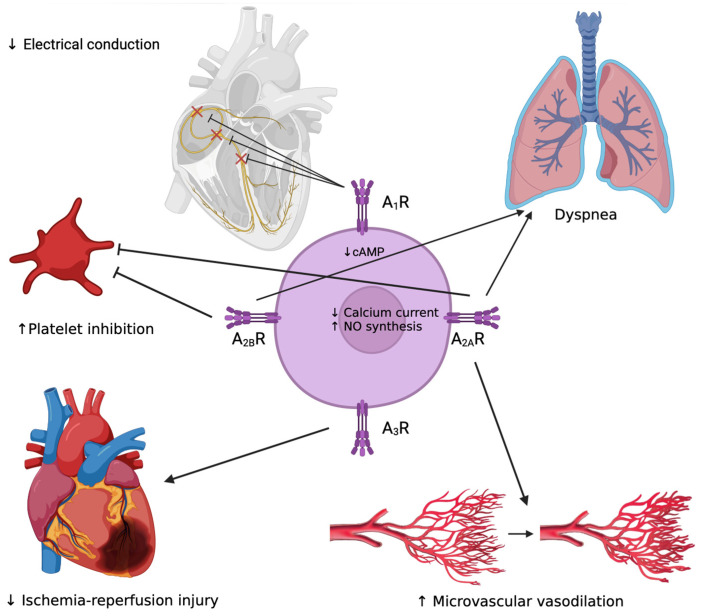
Schematic representation of adenosine effects on conduction system (reduction in electrical conduction through inhibition of SN, AVN and His bundle), platelet activity (inhibition), respiratory system (dyspnea), microvascular function (vasodilation) and ischemia-reperfusion damage (reduction); ↑ enhance; ↓ reduce.

Adenosine effects are mediated by four GPCRs: activation of A_1_R leads to slowing of the heart rate, activation of A_2A_R and A_2B_R leads to vasodilation, and A_3_R is implicated in the protection against the ischemia/reperfusion process [6] (Figure 1).

A_1_R has high affinity for adenosine; its expression is widespread in the cardiovascular system, and its concentration is higher at the level of right atrium [7,8,9]. Its activations lead to a decrease in production of cyclic AMP (cAMP) with the consequent inhibition of protein kinase A (PKA) and voltage-gated calcium channels (VGCCs), the activation of phospholipase C (PLC), and of the inward rectification of IKAdo, Ach currents with a shortening of action potential, duration and refractoriness [3,4,10,11]. Clinically this pathway produces bradycardia, a negative dromotropic effect on the atrioventricular block (AVB) through inhibition of cardiac pacemakers at the level of the sinus node (SN), atrio-ventricular node (AVN) and His bundle [12] and, though modification of action potential, facilitates re-entry mechanisms and atrial arrhythmias [13].

A_2A_R is particularly expressed in the atrium and ventricular tissues, in the vessels and specifically in smooth muscle cells of coronary arteries [7,8,9,14]. Its activation leads to inhibition of VGCCs and the L-type calcium current, stimulation of NO synthesis, and opening of K_ATP_ channels [15,16]. The clinical counterpart of these molecular effects are vasodilatation and coronary blood flow regulation [17,18].

A_2A_R and A_2A_R are expressed on platelets and their activation has antiplatelet effects via inhibition of calcium flux [19].

A_3_R is poorly represented in myocardial tissue but is present in smooth muscle cells of coronary arteries [20]. Its activation is involved in protection from ischemia/reperfusion injury.

### 2.3. Anti-Inflammatory Effects of Adenosine

Adenosine has known anti-inflammatory effects that are the consequence of the interaction with various receptors on immune cells, leading to both pro- and anti-inflammatory responses depending on factors like receptor affinity and cell type. For instance, while low adenosine concentrations activate A_1_R, associated with pro-inflammatory activity, higher concentrations primarily engage other receptors with anti-inflammatory effects, creating a negative feedback loop on the immune system [21,22,23]. Adenosine impacts different immune cells differently; while it inhibits neutrophil adhesion and migration through A_2A_R and A_2B_R, it promotes inflammation in these cells through A_1_R and A_3_R [24,25]. In monocytes/macrophages, adenosine generally exhibits anti-inflammatory effects by reducing secretion of pro-inflammatory cytokines and promoting differentiation in the anti-inflammatory M2 phenotype [26].

Methotrexate (MTX) exerts its anti-inflammatory effects partly through A_2A_R activation, which enhances cholesterol efflux transporter proteins like ATP-binding cassette subfamily A member 1 (ABCA1) and ATP-binding cassette subfamily G member 1 (ABCG1) on macrophages, thereby reducing foam cell formation and atherosclerosis risk. ABCA1 and ABCG1 facilitate cholesterol efflux to form HDL, while cholesterol 27-hydroxylase converts cholesterol into 27-hydroxycholesterol, aiding its removal from cells [27]. Adenosine, via A_2A_R activation, upregulates these transporters and enzymes, preventing lipid overload and foam cell formation [28]. Oxysterols, by activating LXR, further enhance expression of ABCA1 and ABCG1, counteracting inflammatory cytokine-induced suppression of these proteins [29].

Considering the beneficial effects of A_2A_R agonism on cholesterol transport and anti-inflammatory actions, specific A_2A_R agonists could potentially be utilized to treat atherosclerotic cardiovascular disease. Regadenoson is already employed as a vasodilator stress agent, while newer orally administered drugs targeting the A_2A_R may hold promise for future cardio protection medications.

### 2.4. Adenosine in Pre-Conditioning in Myocardial Ischemia

Ischemic preconditioning is defined as a brief transient ischemia before an episode of more prolonged ischemia, which makes the myocardium more resistant to ischemia-reperfusion injury [30]. This process is thought to be mediated by endogenous adenosine through the activation of A_1_R and A_3_R [31,32]. Ischemic preconditioning thorough mechanical occlusion of coronary arteries in patients undergoing coronary artery bypass grafting (CABG) [33] and elective PCI [34] has been shown to improve outcomes in small studies. One small randomized trial of 30 patients showed effective myocardial precondition through 10 min intracoronary adenosine infusion at 2 mg/min vs. normal saline before elective PCI [35]. In the group of patients treated with adenosine there was no difference in ST-segment shift in three subsequent coronary balloon inflations, while in the normal saline group there was a greater ST-segment shift during the first inflation than in the second and third one. Moreover, the ST-segment shift in the adenosine group was significant smaller than in the control group. However, when investigating harder endpoints such as periprocedural increase in myocardial specific enzymes, corrected TIMI frame count and in-hospital death, periprocedural myocardial infarction and in-hospital urgent target-vessel revascularization, no difference was found between intracoronary administration of high-dose adenosine vs. placebo during elective PCI [36].

### 2.5. Anti-Platelet Effects of Adenosine

Platelets express A_2A_R and A_2B_R, and adenosine binding to A_2A_R stimulates adenylate cyclase with the subsequent increase in platelet intracellular cAMP, which inhibits platelet activation [36]. A major role in platelet activation is played by P2Y_12_ ADP receptor. A recent in vitro study showed an inhibition of ADP-induced platelet activation after administration of selective and non-selective A_2A_R agonists, indicating that adenosine receptor agonists could be a potential therapeutic option to enhance P2Y_12_ inhibitor activity [37].

## 3. The Role of Adenosine in Anti-Platelet Therapy Side Effects

### 3.1. Ticagrelor

Adenosine mediates two of most clinically relevant off-target side effects of ticagrelor and, to lesser extent, of cangrelor, represented by dyspnea and bradyarrhythmias.

Ticagrelor is a nucleoside analogue, non-thienopyridine, reversible blocker of the adenosine diphosphate (ADP) receptor P2Y_12_. It acts as a potent P2Y_12_ inhibitor, and thus it is recommended by the European [38] and American [39] guidelines as a first-line agent, in addition to aspirin, for dual anti-platelet therapy (DAPT) in ACS and after PCI in high-risk situations (such as suboptimal stent deployment or other procedural characteristics associated with high risk of stent thrombosis, complex left main stem, or multivessel stenting) in chronic coronary syndromes (CCSs) [40]. The obvious tradeoff of enhancing platelet activity inhibition, compared to less potent P2Y_12_ inhibitors such as clopidogrel or ticlopidine, is represented by the augmented bleeding associated with this drug compared with a less potent P2Y_12_ inhibitor such as clopidogrel (on-target side effect).

As stated previously, adenosine is a product of ADP metabolism and in normal circumstances it is released in the extracellular space and transferred by specific carriers (ENTs and CNTs) to the intracellular space, where it is rapidly converted to inosine and adenine. Ticagrelor, inhibiting specific ENTs, leads to an increase in adenosine concentration in the extracellular space and to its subsequent systemic effects.

Dyspnea is the consequence of the activation of A_1_R and A_2A_R in the vagal C fibers on the bronchial wall [41], while bradyarrhythmia is mediated mainly by the activation of A_1_R.

Dyspnea and bradyarrhythmias are usually temporary and tend to self-resolve in a few days because, in the context of acute myocardial infarction, ischemic myocardial cells also release adenosine, contributing to an increase in circulating adenosine levels causing adenosine overload. However, once myocardial perfusion is restored, adenosine levels tend to normalize [42].

The incidence of dyspnea in patients treated with ticagrelor varies in different studies, from 13.8% in the PLATO trial [43] to 20% in the more recent TWILIGHT trial [44]. Angiolillo et al. [45] analyzed dyspnea in the TWILIGHT trial and found that discontinuation of ticagrelor due to dyspnea occurred in 9.1% patients, in most of the cases (6.3%) within the first 3 months of treatment. In this sub-study, most of the patients that discontinued ticagrelor switched to clopidogrel (76.7%).

Since dyspnea is a relativity common side effect of ticagrelor observed in clinical practice, it is important to confirm that there is a causal relationship with ticagrelor excluding other causes of dyspnea, while monitoring the patient for a few days before switching to another anti-platelet drug. In the event of a switch, genetic testing before or platelet reactivity testing after may be considered [46].

The incidence of bradyarrhythmias related to ticagrelor in the PLATO trial [43] was investigated by Scirica et al. [47]. Ventricular pauses ≥ 3 s occurred in 5.8% of patients (compared to 3.6% in patients treated with clopidogrel) in the first week after randomization to ticagrelor. However, at 1 month their incidence was similar in the two groups (2.1% vs. 1.7%). In the trial, the rate of clinical bradycardic episodes was very low: syncope (0.3% vs. 0.2%), heart block (0.4% vs. 1%), and pacemaker implantation (0.5% vs. 1%).

Although bradyarrhythmias related to ticagrelor are less frequent than dyspnea, their management is more challenging. Real-life cases involving relatively young patients without high bleeding risk (HBR) features, like the one reported by Cesarini et al. [48], should be switched to prasugrel. Meanwhile older patients with one or more HBR features, like the one reported by Di Filippo et al. [49], should be switched to clopidogrel, possibly after the evaluation of the presence of loss-of-function alleles of the CYP2A19 enzyme. In all cases, these patients should be monitored for a few days after the switch while completing the diagnostic workup. Pacemaker implantation should be reserved for patients with pathological findings or a recurrence of bradycardic events.

Some case reports [50,51] documented positive effects on ticagrelor-related bradyarrhythmias and dyspnea with continuous infusion of aminophylline or theophylline, which act as a adenosine receptor antagonists. Currently, no randomized controlled trials have been conducted, and there are no specific recommendations in either the American or European guidelines.

### 3.2. Cangrelor

Cangrelor is an intravenous, nonthienopyridine adenosine triphosphate (ATP) analogue, reversible, and a P2Y_12_ inhibitor, which binds to a different portion of the receptor compared to ticagrelor and it is characterized by a short half-life (3–6 min) and rapid onset of action (within a few seconds, maximal inhibition in 15 min) [52].

Nowadays, many patients are treated with cangrelor and the two main clinical contexts in which it is used are PCI in ACS in P2Y_12_-naïve patients and bridging to high-risk surgery in patients who cannot stop anti-platelet therapy. In the most recent ESC Guidelines [38], cangrelor is recommended (COR IIb, LOE A) for P2Y_12_-naïve patients undergoing PCI for ACS for the duration of the PCI or at least for 2 h. The BRIDGE trial [53] demonstrated the efficacy of intravenous cangrelor in the maintenance of high levels of platelet inhibition in patients discontinuing thienopyridines for cardiac surgery. The aging of the population and the consistent burden of coronary artery disease is leading to a larger proportion of patients with a recently placed stent who need non-deferrable surgery or invasive procedures. Cangrelor allows adequate anti-platelet inhibition during the washout of oral P2Y_12_ inhibitor prior to surgery and a safe resumption of anti-platelet inhibition shortly after the surgery, with the option of suspension in event of bleeding. In this clinical context the administration of cangrelor may be prolonged for days and some authors suggest a tailored dose adjustment based on daily platelet reactivity testing to minimize side effects [54]. In the CHAMPION PHOENIX study, the incidence of dyspnea in patients treated with cangrelor was 1.2% (vs. 0.3% placebo group) and usually mild or moderate, leading to no discontinuation of the therapy [55]. Nevertheless, some authors have reported cases of discontinuation of both ticagrelor and cangrelor because of intense dyspnea [49].

Cangrelor-induced dyspnea has analogous mechanisms to that induced by ticagrelor. The short half-life of cangrelor is related to its rapid catabolism, which produces one main metabolite, the so-called CMM (Cangrelor Main Metabolite). CMM has no inhibitory effects on P2Y_12_ and it is a weak inhibitor of ENT1, leading to an elevation of adenosine extracellular concentration with the potential same effects reported by the adenosine theory [55]. However, the mechanisms underlying the off-target side effects of cangrelor are unclear and, likewise, the reason for their lower incidence compared to ticagrelor.

## 4. The Role of Adenosine in Treatment of the No-Reflow Phenomenon

The no-reflow phenomenon (NRP) is defined as Thrombolysis In Myocardial Infarction (TIMI) Flow Grade (TFG) < 3 and Myocardial Blush Grade (MBG) < 3 in an open coronary artery [56]. TFG is an angiographic index of epicardial perfusion (Table 1) designed by the TIMI Study group to standardize the evaluation of coronary angiographies in the TIMI trial Phase 1, which evaluated the efficacy of intravenous streptokinase vs. tissue plasminogen activator (tPA) in the recanalization of the culprit artery in STEMI patients [57].

MBG is an angiographic measure of myocardial perfusion (Table 2) in patients with restored patency of the infarct-related coronary artery [58] and it is evaluated as contrast density in the myocardial perfused by the coronary artery. It allows the identification of patients who exhibit the no-reflow phenomenon: in these cases, the angiographic contrast medium passes from the arterial system to the venous system through alternative pathways rather than the microcirculation in the infarcted area.

The no-reflow phenomenon is explained by the fact that, following a prolonged coronary occlusion, the microcirculation loses its anatomical integrity, and upon restoration of epicardial flow, myocardial perfusion is only restored in areas where the microcirculation is preserved.

NRP occurs mainly after PCI in the context of ST-segment elevation myocardial infarction (STEMI) or in PCI of degenerated saphenous vein grafts (SFGs) [59]. It is usually associated with ECG ischemic changes (ST-segment deviation) and may be associated with re-occurrence or increase in symptoms and hemodynamic instability [60].

The mechanisms behind this phenomenon are several: microvascular obstruction due to distal embolization of thrombus or debris, microvascular spasm, intravascular plugging from platelet microthrombi or leukocytes, and ischemia-reperfusion injury [56,59,61].

Several clinical risk factors are reported to be associated with NRP, such as female sex, older age, hypertension, diabetes, dyslipidemia, delayed presentation (>6 h) and chronic kidney disease. High thrombus burden is the main angiographic risk factor [56,60,61].

Preventive strategies are mainly the avoidance of high-pressure post-dilatation, thrombus aspiration in the case of high thrombus burden to prevent distal embolization and, when using atherectomy, short burr run, lower burr speed and avoidance of deceleration. In SVGs PCI pretreatment with intracoronary vasodilators and the use of coronary filter should be considered [56].

The treatment of NRP is based on vasodilators such as adenosine, nitroprusside and non-dihydropyridine calcium channel blockers.

The first studies investigating the effects of adenosine on outcomes following PCI for STEMI were the AMISTAD (Acute Myocardial Infarction Study of Adenosine) trial [62] and AMISTAD-II trial [63]; these trials demonstrated a significant reduction in infarct size with infusion of high-dose adenosine, with no benefit with respect to clinical outcomes (death, congestive heart failure and re-hospitalization for congestive heart failure). A separate analysis of the AMISTAD-II trial showed that adenosine was linked to better clinical outcome in patients who underwent early reperfusion [64].

The REOPEN-AMI (Intracoronary Nitroprusside Versus Adenosine in Acute Myocardial Infarction) trial examined the effects of adenosine vs. sodium nitroprusside infusion in STEMI patients following thrombus aspiration and found a significant better ST-segment resolution after 90 min in the adenosine group, a favorable ventricular remodeling at 1 year (but no difference at 30 days) and a lower incidence of a composite endpoint of death, myocardial infarction, and heart failure [62].

The recently published COAR trial [65] compared epinephrine and adenosine in treatment of NRP, showing a better improvement in the coronary flow with epinephrine (more TFG > 3 and better corrected TIMI frame count), with no significant difference in the final Myocardial Blush Grade 3, and with a mean reduction in corrected TIMI frame count, in-hospital and short-term mortality, and major adverse cardiac events.

Meta-analyses showed conflicting results: in two of them, adenosine infusion reduced post-procedural no-reflow, with no benefits in term of mortality, re-myocardial infarction and ST-segment resolution [66,67], while in another a favorable result appeared, with a significant increase in left ventricular function, a reduction in incidence of heart failure and a lower incidence of major adverse cardiovascular events (MACEs) [68].

## 5. Diagnosis and Treatment of Supraventricular Arrythmias

Adenosine administration at incremental bolus of 6 mg, 12 mg and 18 mg followed by rapid flush of normal saline is the first-line and usually efficacious therapy for treatment of supraventricular tachycardias involving the AVN, such as typical AV nodal re-entrant tachycardia, AV reciprocating tachycardia with a concealed accessory pathway, and AV reciprocating tachycardia in Wolff–Parkinson–White syndrome.

From a diagnostic point of view, adenosine, causing transient AVB, can reveal atrial flutter or atrial fibrillation and aid in the differential diagnosis in broad complex tachycardias [69]. These effects are mainly mediated by A_1_R.

## 6. Adenosine in Diagnosis of Coronary Artery Disease

### 6.1. Adenosine Analogues

Adenosine, dipyridamole, and regadenoson are used as equivalent coronary vasodilators during cardiac stress testing [70]. The rationale of these stress tests is the “steal phenomenon”: the administration of a vasodilator leads to an increase in blood flow that favors non-diseased coronary arteries at the expense of diseased vessels.

Dipyridamole is an indirect coronary-artery vasodilator that inhibits the nucleoside transporter responsible for the cellular uptake of adenosine. This transporter explains the extremely short half-life of adenosine, and its blockade by dipyridamole results in greater extracellular levels of adenosine and enhancement of its actions.

Regadenoson is a direct coronary-artery vasodilator, and it is a selective low-affinity A_2A_R agonist that mimics the effects of adenosine in causing coronary vasodilatation and increasing myocardial blood flow. It is a very weak agonist of the A_1_R. Furthermore, it has negligible affinity to A_2B_R and A_3_R.

### 6.2. Cardiac Stress Imaging

The main non-invasive stress testing modalities are represented by an echocardiogram, gadolinium-enhanced cardiac magnetic resonance imaging (CMR) and positron emission tomography (PET), or single-photon emission computed tomography (SPECT) with infusion of a radiotracer (e.g., Tc-99m).

The pharmacological stress echocardiography is performed under continuous ECG and regular pressure cuff monitoring and aims to identify new regional wall motion abnormalities (not present on the baseline echocardiogram) with or without ischemic ST-segment changes or symptoms. The induction of hypokinesia or akinesia during pharmacological stress in three or more segments (in a 16-segment model) confers a high event risk (cardiac mortality rate > 3% per year) [71].

The stress CMR with gadolinium infusions aims to evaluate rest and stress global left ventricular function and regional wall abnormalities, along with the presence of late gadolinium enhancement (LGE). The presence of stress perfusion defects in two or more segments (in a 16-segment model) confers a high event risk [71].

The perfusion PET/SPECT aims to detect fixed or reversible perfusion defects: when the radiotracer is administered during vasodilator infusion, in the presence of epicardial coronary stenosis, a perfusion defect is observed, and delayed images then show if the defect is reversible [72]. An area of ischemia involving 10% or more of the left ventricle myocardium is considered to be at high risk of events [71].

### 6.3. Intracoronary Physiological Assesment

The latest ESC guidelines on ACS and CCS [38,40] and many expert consensus documents support the use of coronary physiological assessment to guide PCI, in particular in angiographically intermediate coronary stenosis.

Almost 30 years ago it was demonstrated that, under the condition of maximal hyperemia, the ratio of the pressure distal to the coronary stenosis to the aortic pressure was linear, and correlated with the ratio of the Doppler-derived flow velocity [73]. Technological progress made it possible to incorporate miniaturized pressure sensors in coronary guidewires, allowing relatively easy direct intracoronary pressure measurements.

Sensor-tip guidewires allow the assessment of the functional significance of intermediate coronary stenosis and the function of coronary microcirculation.

The functional significance of epicardial stenosis can be assessed by hyperemic (Fractional Flow Reserve, FFR) or non-hyperemic (e.g., Pd/Pa or instantaneous wave-Free Ratio, iFR) indexes. Both types of indexes are calculated as the ratio of the pressure distal to the coronary stenosis and the pressure proximal to the coronary stenosis (which is considered to be equal to the aortic pressure) under rest or hyperemia.

A hyperemic condition can be induced by administration of adenosine as continuous intravenous infusion (140 mg/kg/min) or intracoronary bolus (200 µg for the left coronary artery, 100 µg for the right coronary artery), of regadenoson administered as rapid intravenous bolus (400 µg in 10 s), papaverine as intracoronary bolus (15 mg for the left coronary artery, 10 mg for the right coronary artery) or contrast media. Continuous intravenous adenosine infusion is usually preferred over intracoronary bolus because it allows for more reproducible measurements and longitudinal pull-back [74].

In recent years, there has been a growing interest in patients with angina and/or ischemia with non-obstructive coronary artery disease (ANOCA and/or INOCA), leading to a “full physiology” coronary assessment approach [75]. After assessing the significance of epicardial coronary stenosis, the coronary microcirculation is evaluated through measurements of the Coronary Flow Reserve (CFR) and the Index of Microvascular Resistance (IMR) measurement, both indexes needing to be measured during hyperemia. The impairment of these indexes, either individually or in combination, allows for the classification of patients into specific subtypes of microvascular dysfunction, thereby guiding the selection of appropriate pharmacological treatments [74].

## 7. Adenosine Side Effects

Adenosine is usually well tolerated and its side effects, like its diagnostic/therapeutic effects, are short, due to its very short half-life (<10 s). Side effects are more frequent when adenosine is administered intravenously rather than intracoronally. Adenoscan registry [76] aimed to determine the safety of intravenous adenosine infusion (at 140 µg/kg/min) during radionuclide imaging, and the reported side effects were bronchospasm (0.1%), arrythmias (3.3%), AV block (7.6%), headache (11%), gastrointestinal discomfort (14%), chest pain (34.6%), dyspnea (35.2%) and flushing (36.5%). In this observational study, the side effects were more common in female and younger patients and patients with a higher weight (≥82 kg for men and (≥72 kg for women). In another registry of patients undergoing MRI stress testing with adenosine, the incidence of side effects was lower (chest pain and dyspnea in 14% and nausea and vomiting in 5%); these different results can be explain by the lower dose of adenosine used in this protocol [77].

Adenosine, like other anti-arrhythmic drugs, has pro-arrhythmic side effects. Atrial fibrillation during adenosine infusion is reported in 12% of patients [78] and it is usually well tolerated in the absence of an accessory pathway, which can induce a high ventricular rate response which can degenerate in ventricular fibrillation. Ventricular arrythmias are also reported and are usually associated with bradycardia.

## 8. Conclusions

Adenosine is an endogenous purine and an exogenous drug of interest for the interventional cardiologist and cardiologist involved in CAD management, both for its role in the adverse effects of drugs frequently used and for its use in diagnostic and therapeutic scenarios. Besides its effects on coronary circulation vascular tone, it has anti- and pro-arrhythmic potential, along with anti-inflammatory effect and a potentially protective role against ischemia-reperfusion injury.

## 9. Future Directions

Future research should focus on further understanding the subtype-specific roles of adenosine receptors in cardiovascular disease to allow the developing of selective agonists or antagonists tailored to modulate specific aspects of cardiac function, thereby minimizing off-target effects and optimizing therapeutic efficacy.

The role of adenosine in ischemic preconditioning should be further investigated, as it can find application in the prevention of ischemia-reperfusion injury after myocardial infarction or cardiac surgery.

Some evidence [79] suggests that adenosine signaling pathways play a critical role in regulating cardiac remodeling processes, including fibroblast activation, extracellular matrix deposition, and inflammation, so this field should be explored as it can have a therapeutic role in the prevention of myocardial fibrosis and maladaptive cardiac remodeling.

## Figures and Tables

**Table 1:** Definition of epicardial coronary perfusion by TIMI Flow Grade.

TIMI Flow Grade	
TFG 0	Absence of antegrade flow beyond the point of occlusion.
TFG 1	The contrast passes beyond the occlusion but fails to opacify the entire coronary bed distal to the obstruction.
TFG 2	The contrast passes beyond the occlusion but fails to opacify the entire coronary bed distal to the obstruction, but the rate of its entry or clearance is significantly slower than comparable areas not perfused by the previously occluded vessel (e.g., another coronary artery).
TFG 3	Antegrade flow into the bed distal to the obstruction occurs as promptly as antegrade flow into the bed proximal to the obstruction, and clearance of contrast material from the involved bed is as rapid as clearance from an uninvolved bed in the same vessel or in the opposite artery.

**Table 2:** Definition of myocardial perfusion by Myocardial Blush Grade.

Myocardial Blush Grade	
MBG 0	No myocardial blush
MBG 1	Minimal myocardial blush
MBG 2	Moderate myocardial blush but less than that obtained during angiography of a contralateral or ipsilateral non-infarct-related coronary artery
MBG 3	Normal myocardial blush, comparable with that obtained during angiography of a contralateral or ipsilateral non-infarct-related coronary artery

## Data Availability

Not applicable.
